# Systematic identification and validation of the reference genes from 60 RNA-Seq libraries in the scallop *Mizuhopecten yessoensis*

**DOI:** 10.1186/s12864-019-5661-x

**Published:** 2019-04-11

**Authors:** Yajuan Li, Lingling Zhang, Ruojiao Li, Meiwei Zhang, Yangping Li, Hao Wang, Shi Wang, Zhenmin Bao

**Affiliations:** 1 MOE Key Laboratory of Marine Genetics and Breeding, Ocean University of China, 5 Yushan Road, Qingdao, China; 2 Laboratory for Marine Fisheries Science and Food Production Processes, Qingdao National Laboratory for Marine Science and Technology, Qingdao, China; 3 Laboratory for Marine Biology and Biotechnology, Qingdao National Laboratory for Marine Science and Technology, Qingdao, China

**Keywords:** Reference genes, Transcriptome-wide, Scallop, Early development, Adult tissues, Gonadal development

## Abstract

**Background:**

Reverse transcription quantitative PCR (RT-qPCR) is widely used for gene expression analysis in various organisms. Its accuracy largely relies on the stability of reference genes, making reference gene selection a vital step in RT-qPCR experiments. However, previous studies in mollusks only focused on the reference genes widely used in vertebrates.

**Results:**

In this study, we conducted the transcriptome-wide identification of reference genes in the bivalve mollusk *Mizuhopecten yessoensis* based on 60 transcriptomes covering early development, adult tissues and gonadal development. A total of 964, 1210 and 2097 candidate reference genes were identified, respectively, resulting in a core set of 568 genes. Functional enrichment analysis showed that these genes are significantly overrepresented in Gene Ontology (GO) terms or Kyoto Encyclopedia of Genes and Genomes (KEGG) pathways related to ribosomes, energy production, etc. Six genes (*RS23*, *EF1A*, *NDUS4*, *SELR1*, *EIF3F*, and *OLA1*) were selected from the candidate genes for RT-qPCR validation, together with 6 commonly used reference genes (*ACT, CYTC, HEL, EF1B, GAPDH* and *RPL16*). Stability analyses using geNorm, NormFinder and the comparative delta-Ct method revealed that the new candidate reference genes are more stable than the traditionally used genes, and *ACT* and *CYTC* are not recommended under either of the three circumstances. There was a significant correlation between the Ct of RT-qPCR and the log_2_(TPM) of RNA-Seq data (Ct = − 0.94 log_2_(TPM) + 29.67, R^2^ = 0.73), making it easy to estimate the Ct values from transcriptome data prior to RT-qPCR experiments.

**Conclusion:**

Our study represents the first transcriptome-wide identification of reference genes for early development, adult tissues, and gonadal development in the Yesso scallop and will benefit gene expression studies in other bivalve mollusks.

**Electronic supplementary material:**

The online version of this article (10.1186/s12864-019-5661-x) contains supplementary material, which is available to authorized users.

## Background

Reverse transcription quantitative PCR (RT-qPCR) is the most frequently used method in gene expression analysis due to its high sensitivity, specificity, reproducibility and broad dynamic range [1]. To obtain reliable RT-qPCR results, many considerations need to be taken [2]. One essential component of a reliable RT-qPCR assay is normalization, which controls for variations in the amount and quality of RNA between different samples, thus enabling comparisons of the expression of a gene of interest among different samples. Reference genes are most commonly used for normalization. Choosing a reference gene that shows stable expression across all samples is critical for relative quantification in RT-qPCR assays.

Despite the awareness of the importance of reference genes, most previous studies directly applied commonly used reference genes for gene expression quantification without validation or conducted reference gene selection from a few traditionally used reference genes [3–6]. However, an increasing number of recent studies have shown that the frequently used reference genes are not always stable under different conditions [7, 8]. Housekeeping genes, which maintain constant expression levels in all cells and under all conditions, are an ideal source for reference gene selection. In mammals, housekeeping genes have been detected using various large-scale gene expression profiling methods, such as expressed sequence tags (ESTs), serial analysis of gene expression (SAGE) and microarray analysis [9–11]. The advent of RNA-Seq enables the identification of housekeeping genes in well-studied model organisms and less-studied nonmodel organisms. To date, housekeeping or reference genes have been successfully identified from transcriptome data in various organisms, such as humans [12], mice [13], zebrafish [14], tomato [15], seaweed [16] and kiwi [17].

Bivalve mollusks comprise approximately 14,000 existing species that are widely distributed worldwide. Many of these species are economically important aquaculture shellfish; therefore, extensive studies on the genes related to immunity [18–20], growth [21–23] and reproduction [24, 25] have been conducted in this group of animals. Gene expression quantification via RT-qPCR has been extensively conducted, but mostly using a single reference gene, such as *ACT* and *CYTC* [26–28]. To eliminate misleading effects due to the use of inappropriate reference genes, reference genes have been selected but generally focused on some traditionally used genes [29–31]. To our knowledge, the transcriptome-wide identification of reference genes has only been conducted in the Pacific oyster [32, 33], despite the availability of extensive transcriptome data in various organisms [34–38].

In this study, we carried out systematic analyses of the 60 transcriptomes covering early development, adult tissues and gonadal development in the Yesso scallop *M. yessoensis*. The candidate reference genes were identified for each dataset, and a core set was generated. Six selected candidate reference genes were compared with 6 commonly used reference genes for the RT-qPCR validation of stability, and the relationship between the Ct of the RT-qPCR and transcripts per million (TPM) of the RNA-Seq was examined. Our study provides a valuable resource of the reference genes for the scallop. This information will contribute to the accurate quantification of gene expression in future studies.

## Methods

### Sample collection

The Yesso scallops used for RT-qPCR were sampled from the hatchery of the Zhangzidao Group Co., Ltd. (Dalian, China).

Embryos (two to eight cells, blastulae, and gastrulae) and larvae (trochophore larvae, D-stage larvae, pediveliger larvae, and juvenile) were obtained as described in Sun et al. [39] and Wang et al. [34]. Then, the samples were preserved in RNAlater and stored at − 80 °C until further use.

Two-year-old adult scallops were maintained in aerated seawater for a week. After acclimation, the following tissues were dissected: digestive gland, eyes, foot, gill, gonad, hemocytes, mantle, striated muscle, smooth muscle and nerve ganglia. The ovaries and testes of mollusks at four developmental stages (resting stage, proliferative stage, growing stage and maturation stage) were collected as described in Li et al. [40]. Three biological replicates were collected for each tissue, frozen in liquid nitrogen, and then stored at − 80 °C prior to RNA isolation.

### RNA-Seq datasets

To identify reference genes in early development, the RNA-Seq data from 7 embryo/larva stages (two to eight cells, blastulae, gastrulae, trochophore larvae, D-stage larvae, pediveliger larvae and juvenile) were obtained from Wang et al. (NCBI Bioproject ID: PRJNA259405) [34]. Twenty-nine RNA-Seq datasets for the eyes, mantle, gill, hemocytes, digestive gland, striated muscle, smooth muscle, foot, ganglia, ovaries and testes (NCBI Bioproject ID: PRJNA259405 and PRJNA423107) [34, 36, 41] were used to identify the reference genes across tissues. Reference genes during gonadal development were evaluated using 24 RNA-Seq datasets from four developmental stages of ovaries and testes (NCBI Bioproject ID: PRJNA516336) [42]. Detailed information on all RNA-Seq data is available in Additional file 1: Table S1.

### Data quantification and analysis

All raw reads downloaded from NCBI (https://www.ncbi.nlm.nih.gov) were first filtered using a homemade Perl script to obtain high-quality reads by removing reads containing undetermined bases (‘N’) or excessive numbers of low-quality positions (> 10 positions with quality scores < 20). Then the high-quality reads were mapped to the *M. yessoensis* genome [34] using STAR [43] with the parameters of ‘STAR --runThreadN 8 --genomeDir --readFilesIn R1.fq R2.fq --sjdbGTFtagExonParentGene --outFilterMultimapNmax 100000 --outFileNamePrefix --outReadsUnmapped Fasta >STAR_sample.log’. The raw counts for each gene were converted into TPM using the RNA-Seq by Expectation Maximization (RSEM) software package.

### Identification of reference genes from transcriptomes

The reference genes were detected using the method provided by Eisenberg and Levanon [12], with minor modifications. Briefly, considering that RPKM is not appropriate for comparisons between samples [44–46], we used TPM values to measure gene expression levels. After calculating the TPM values for each gene in all samples, TPMs from biological replicates were averaged for subsequent analysis. Finally, four criteria were adopted for the detection of reference genes: (I) expression observed in all tissue types or developmental stages; (II) low variance over tissues or developmental stages by requiring standard-deviation [log_2_(TPM)] < 1; (III) no exceptional expression in any single tissue or developmental stage by requiring no log_2_(TPM) differed from the mean log_2_(TPM) by two or more; and (IV) medium to high expression level by requiring mean [log_2_(TPM)] > 5. The stability of the candidate reference genes was further evaluated according to the coefficient of variation (CV = stdev/mean). Here, all candidate reference genes have a mean [log_2_(TPM)] > 5 and a standard-deviation [log_2_(TPM)] < 1, so their CV values are always less than 0.2.

### Functional enrichment analysis

To better understand the function of the reference genes, GO and KEGG enrichment analyses were performed. The Swiss-Prot Blast results for all genes were imported into Blast2GO, and GO terms at level 3 were assigned to produce a broad overview of the groups of genes for biological processes, cellular component, and molecular function categories. The KEGG annotation was conducted by the KEGG automatic annotation server (KAAS). The significance of GO and KEGG enrichment was determined by using the algorithm implemented in GOstats with an FDR cutoff of 0.05 [47].

### RNA extraction and cDNA synthesis

For all samples, total RNA was extracted using the conventional guanidinium isothiocyanate method, and potential DNA contamination was eliminated by DNase I (Takara Bio, Shiga, Japan) digestion. The obtained RNA samples were then assessed on 1.5% agarose gel electrophoresis and determined by using Nanovue Plus (GE Healthcare, Piscataway, United States) to detect the RNA concentration and purity. cDNA synthesis was carried out using MMLV Reverse Transcriptase (TaKaRa Bio, Shiga, Japan) and oligo(dT)_18_. Finally, the cDNA products were diluted 20-fold with nuclease-free water for RT-qPCR and kept at − 20 °C until further use.

### RT-qPCR validation

Twelve genes, including 6 new candidate and 6 commonly used reference genes, were selected for RT-qPCR validation. Their primers were designed using Primer Premier 5.0 software. Two additional criteria need to be met, including PCR products between 80 and 120 bp in size and an annealing temperature of approximately 63 °C. The primer sequences are listed in Table 1. The amplification efficiency of each primer pair was calculated based on the standard curve generated from a two-fold dilution series of cDNA spanning eight orders of magnitude (0.78 ng ~ 100 ng).

**Table 1:** List of primers used for RT-qPCR analysis

Gene ID	Accession No.	Gene Name	Primer Sequence (5′-3′)	Amplicon Length (bp)	Amplification efficiency
PYT16215	XM_021518643.1	RS23	F:TTACACGAATATCCGCCATCA	100	1.05
			R:GTAATCGTTATCGTGCCACTT		
PYT23375	XM_021500266.1	EF1A	F:GCGGTGGTATTGACAAGAGA	113	1.04
			R:GTTCACGTTCAGCCTTCAGT		
PYT15332	XM_021490269.1	NDUS4	F:TGTGAGAAGCTGGTGTGCTA	108	1.00
			R:TGTGTCTTCCGTCCATTCTAT		
PYT08134	XM_021506528.1	SELR1	F:AAGGCTGGATCAGCGTACTT	97	1.04
			R:CCGTTCTCACACGCCTTAAT		
PYT12324	XM_021496512.1	EIF3F	F:TGCTTCGCTGTGCCTCATAA	105	1.04
			R:TGACTTCAGCTGCATTGACTT		
PYT24193	XM_021485429.1	OLA1	F:AAGGTCAAGGTCTCGGCAAT	100	1.02
			R:CACGTGGACAATCTCTTCGT		
PYT04221	XM_021511578.1	ACT	F:GACAGCTACGTAGGAGATGA	112	1.02
			R:TGATGCCAGATCTTCTCCATA		
PYT19618	XM_021517608.1	CYTC	F:TGTATGTGACAGTCCTTGGTT	126	1.01
			R:GTGTGGCATTGAGCACACTT		
PYT19918	XM_021485125.1	HEL	F:CAGGAAGCAGTGGACTTACA	100	1.02
			R:TCATAACGGTCACCGTAAGAA		
PYT19808	XM_021522100.1	EF1B	F:TAGGCCAGTATGGACCTTCA	105	1.05
			R:TCTTCTTCCTCTTCAGAGTCT		
PYT21544	XM_021497981.1	GAPDH	F:GTGTACATGCTGAAGTACGAT	115	1.04
			R:CGCTCCATGAAGACAGAGAT		
PYT03203	XM_021484929.1	RPL16	F:GTTATCTTCAGTACGCTCACA	103	1.00
			R:ACGCCATGGATATTCTATCCT		

RT-qPCR reactions were conducted in a 384-well plate using a Light Cycler 480 Real-time PCR System (Roche Diagnostics, Mannheim, Germany). Each reaction contained 2 μl 20-fold diluted cDNA, 10 μl Light Cycler 480 SYBR Green I Master and 4 μl 2 μM primers. The cycling conditions were 95 °C for 10 min, followed by 40 cycles of 95 °C for 15 s and 63 °C for 1 min. Each RT-qPCR analysis was performed in triplicate. For each gene, the melting curve and gel picture were analyzed to confirm that a single PCR product had been amplified.

The expression stabilities of the twelve reference genes were assessed using three statistical approaches, geNorm [48], NormFinder [49], and comparative delta-Ct method [50]. The geometric mean was calculated for the ranking values in the three methods to guarantee a comprehensive analysis.

## Results

### Identification of the candidate reference genes from RNA-Seq data

**Fig. 1:**
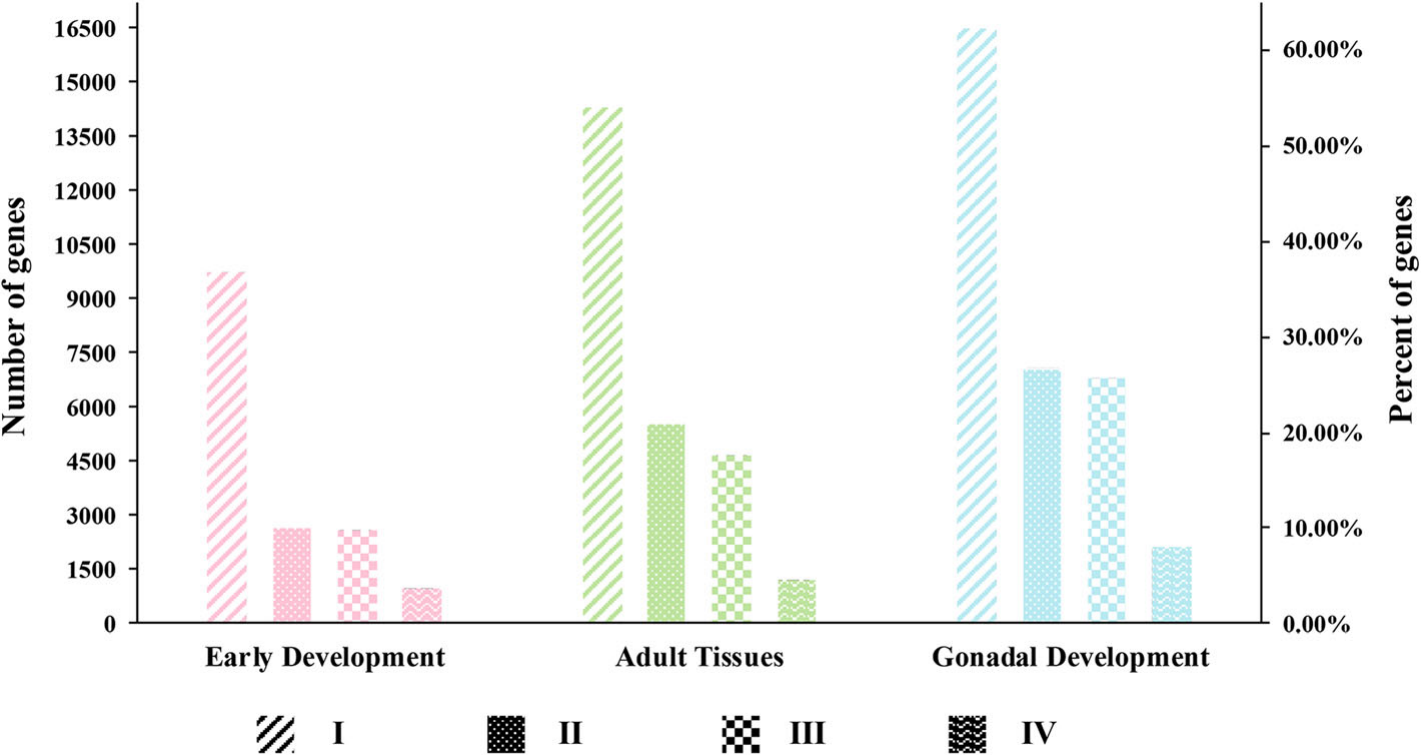
Results of each screening procedure for all three datasets. Both the number and percentage of genes that met the four criteria are shown. (I) TPM > 0; (II) standard-deviation [log_2_(TPM)] < 1; (III) no log_2_(TPM) differed from the mean log_2_(TPM) by two or more; (IV) mean [log_2_(TPM)] > 5. Different colors represent different datasets, pink represents early development, green represents adult tissues and blue represents gonadal development.

**Fig. 2:**
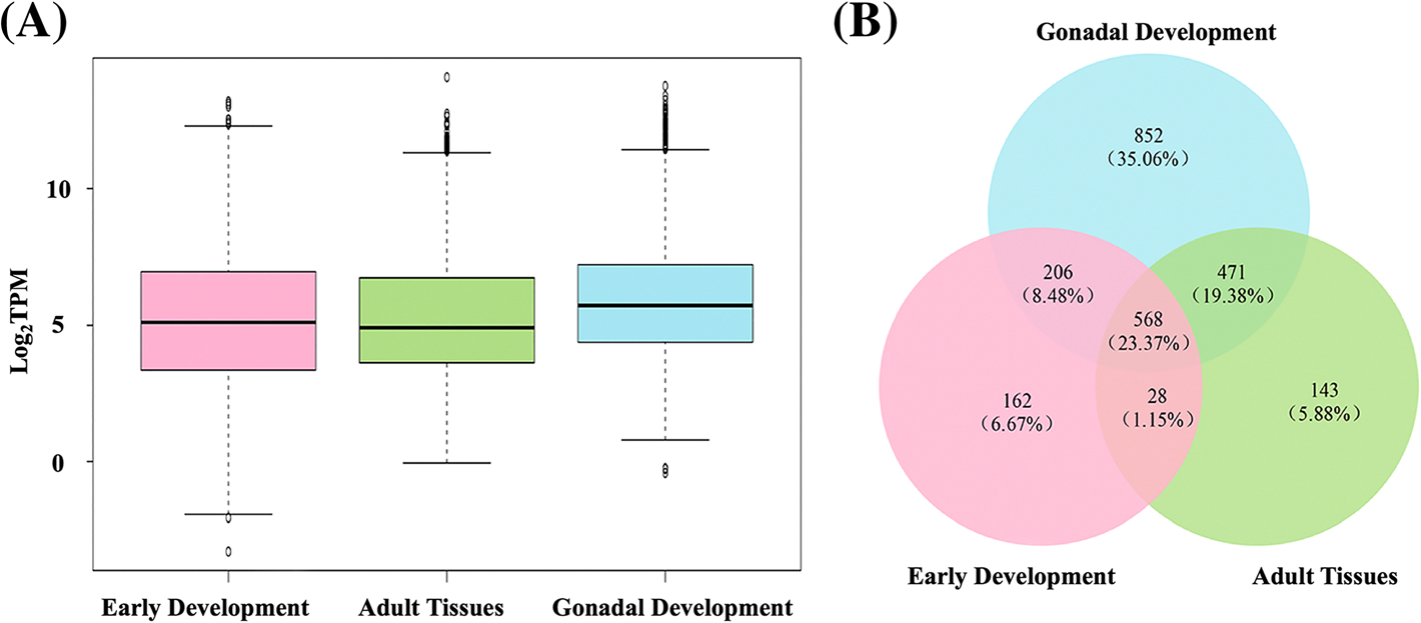
Information on the stable genes in early development, adult tissues and gonadal development. **a** Boxplot showing the log_2_(TPM) for genes that passed criteria I to III in the three datasets. **b** A Venn diagram showing the relationships of candidate reference genes that passed criteria I to IV in the three datasets. Pink: early development; Green: adult tissues; Blue: gonadal development

A total of 60 RNA-Seq datasets from scallop embryos/larvae and adult tissues were used to select candidate reference genes for early development, adult tissues and gonadal development. As shown in Fig. 1, among the 26415 genes in the scallop genome, 2560 (9.69%), 4638 (17.56%) and 6780 (25.67%) genes met the first three filter criteria. To obtain the medium expression level for the fourth criterion, we investigated the expression levels of the genes screened by the first three steps and found that the median log_2_(TPM) values for these genes are approximately 5 for all three datasets (Fig. 2a). Thus, a final criterion was applied to obtain reference genes with an average log_2_(TPM) value higher than 5, resulting in 964 (3.65%), 1210 (4.58%) and 2097 (7.94%) candidate reference genes for early development, adult tissues, and gonadal development, respectively (Additional file 2: Table S2). The 10 most stable genes with the lowest CV values in early development, adult tissues, and gonadal development are listed in Table 2. According to the results, the ten most stable genes in early development have log_2_(TPM) values ranging from 5.48 to 10.72 and CV values between 0.016 and 0.027. Six genes were annotated, of which four (*ATPK*, *ATP5J*, *ATP5L* and *NDUAB*) encode proteins related to energy production. In contrast, the 10 most stable genes in adult tissues possess higher expression levels, with log_2_(TPM) values ranging from 11.15 to 12.08. All of these genes were annotated as ribosomal proteins. The ten most stable genes in gonadal development showed a wide range of expression, with log_2_(TPM) values ranging from 5.76 to 13.74. All of these genes were annotated, of which 5 (*RS23*, *EF1A*, *RLA1*, *IF4A1* and *RM54*) function in protein synthesis.

**Table 2:** The top 10 candidate reference genes in the early development, adult tissues and gonadal development of the scallop *M. yessoensis*

	Gene ID	Gene Symbol	Gene Name	CV	Mean
Early Development	PYT09804	–	–	0.016	9.68
	PYT05988	ATPK	Putative ATP synthase subunit f	0.024	10.65
	PYT22252	ATP5J	ATP synthase-coupling factor 6	0.025	10.58
	PYT08134	SELR1	Sel1 repeat-containing protein 1	0.025	5.48
	PYT07181	–	–	0.025	8.22
	PYT20584	TBB4B	Tubulin beta-4B	0.026	7.88
	PYT12567	–	–	0.026	10.23
	PYT01876	ATP5L	ATP synthase subunit g	0.026	10.72
	PYT08398	NDUAB	NADH dehydrogenase [ubiquinone] 1 alpha subcomplex	0.027	8.83
	PYT17204	–	–	0.027	10.60
Adult Tissues	PYT25527	RL21	60S ribosomal protein L21	0.030	12.08
	PYT22182	RL40	Ubiquitin-60S ribosomal protein L40	0.031	11.85
	PYT16215	RS23	40S ribosomal protein S23	0.032	11.15
	PYT05179	RS2	40S ribosomal protein S2	0.032	11.38
	PYT03250	RL8	60S ribosomal protein L8	0.032	11.60
	PYT05293	RS24	40S ribosomal protein S24	0.032	11.56
	PYT21874	RS20	40S ribosomal protein S20	0.032	11.81
	PYT12227	RS3	40S ribosomal protein S3	0.032	11.54
	PYT19800	RL27	60S ribosomal protein L27	0.033	11.73
	PYT23759	RL19	60S ribosomal protein L19	0.033	11.89
Gonadal Development	PYT16215	RS23	40S ribosomal protein S23	0.012	11.30
	PYT23375	EF1A	Elongation factor 1-alpha	0.013	13.74
	PYT15332	NDUS4	NADH dehydrogenase [ubiquinone] iron-sulfur protein 4	0.014	8.51
	PYT24062	RLA1	60S acidic ribosomal protein P1	0.017	13.36
	PYT20779	IF4A1	Eukaryotic initiation factor 4A-I	0.018	10.28
	PYT10011	LARK	RNA-binding protein lark	0.018	9.31
	PYT14375	SMD1	Small nuclear ribonucleoprotein Sm D1	0.019	10.89
	PYT01777	RM54	39S ribosomal protein L54	0.019	5.76
	PYT24327	CSN8	COP9 signalosome complex subunit 8	0.019	7.23
	PYT10755	CYP1	Peptidyl-prolyl cis-trans isomerase 1	0.020	9.86

### Generation and functional enrichment analysis of the core set of reference genes

To obtain a core set of reference genes, we compared the three candidate reference gene sets. As shown in Fig. 2b, the candidate reference genes identified from different datasets resulted in a total of 2430 genes, of which 568 (23.37%) were shared. GO enrichment analysis (Table 3) revealed that the 568 genes were highly overrepresented in biological process (BP) terms associated with biosynthetic process (FDR = 1.07E-34) and cellular metabolic process (FDR = 4.43E-32) and in molecular functions (MF) terms related to structural constituent of ribosome (FDR = 4.73E-252). These genes were also enriched in cellular components (CC) terms associated with various protein complexes and organelles. KEGG pathway enrichment analysis (Table 3) revealed that the core set reference genes was significantly enriched in 6 pathways, with ribosome (FDR = 5.41E-197) being the most significant, followed by oxidative phosphorylation (FDR = 1.20E-54).

**Table 3:** GO terms and KEGG pathways enriched in the core reference genes of the scallop *M. yessoensis*

ID code	Term/Pathway	Gene Number	*P* value	FDR
GO				
GO:0009058	biosynthetic process	119	2.64E-36	1.07E-34
GO:0044237	cellular metabolic process	184	1.18E-33	4.43E-32
GO:0009056	catabolic process	24	1.48E-03	6.50E-03
GO:0045184	establishment of protein localization	17	3.45E-03	1.38E-02
GO:0030529	ribonucleoprotein complex	83	1.77E-218	4.30E-216
GO:0043228	nonmembrane-bounded organelle	89	5.81E-95	5.14E-93
GO:0043234	protein complex	67	8.58E-34	3.34E-32
GO:0016469	proton-transporting two-sector ATPase complex	13	1.12E-13	1.95E-12
GO:0043227	membrane-bounded organelle	56	2.46E-12	3.86E-11
GO:0044455	mitochondrial membrane part	9	8.59E-11	1.06E-09
GO:0003735	structural constituent of ribosome	81	4.85E-255	4.73E-252
GO:0004129	cytochrome-c oxidase activity	5	1.48E-07	1.33E-06
GO:0051540	metal cluster binding	10	1.73E-07	1.52E-06
GO:1901363	heterocyclic compound binding	106	2.72E-04	1.43E-03
KEGG				
map03010	Ribosome	87	2.89E-199	5.41E-197
map00190	Oxidative phosphorylation	58	3.20E-56	1.20E-54
map03050	Proteasome	25	2.22E-24	5.94E-23
map03013	RNA transport	25	3.32E-06	6.90E-05
map04141	Protein processing in endoplasmic reticulum	21	1.15E-05	2.14E-04
map00020	Citrate cycle (TCA cycle)	7	3.26E-03	4.06E-02

### Comparisons between candidate and commonly used reference genes by RNA-Seq analysis and RT-qPCR validation

To compare the stability between the candidate and commonly used reference genes, 6 candidate and 6 commonly used reference genes were selected for further analysis. All six candidate reference genes were selected from the core set of reference genes. Four of these genes were chosen from the top 10 candidates shown in Table 2, including *RS23*, *EF1A*, *NDUS4*, and *SELR1*. The other two genes, *EIF3F*, and *OLA1*, were selected from the less stable candidates. The 6 commonly used reference genes include *ACT*, *CYTC*, *HEL*, *EF1B*, *GAPDH* and *RPL16* (Table 4).

**Table 4:** Detailed information on the six selected candidate reference genes and the six commonly used reference genes

Gene ID	Gene Symbol	Gene Name	Embryos/Larvae Development		Adult Tissues		Gonadal Development	
			CV	Mean	CV	Mean	CV	Mean
PYT16215	RS23	40S ribosomal protein S23	0.037	11.66	0.032	11.15	0.012	11.30
PYT23375	EF1A	Elongation factor 1-alpha	0.028	12.43	0.033	12.75	0.013	13.74
PYT15332	NDUS4	NADH dehydrogenase [ubiquinone] iron-sulfur protein 4	0.052	8.24	0.069	7.79	0.014	8.51
PYT08134	SELR1	Sel1 repeat-containing protein 1	0.025	5.48	0.098	5.04	0.032	5.74
PYT12324	EIF3F	Eukaryotic translation initiation factor 3 subunit F	0.035	8.13	0.050	7.80	0.027	8.75
PYT24193	OLA1	Obg-like ATPase 1	0.051	6.87	0.042	6.96	0.057	7.07
PYT04221	ACT	Beta-actin	0.523	8.29	0.223	12.45	0.065	10.08
PYT19618	CYTC	Cytochrome c	0.209	9.33	0.106	8.33	0.181	6.96
PYT19918	HEL	DEAD-box RNA helicase	0.277	3.98	0.163	4.79	0.058	6.37
PYT19808	EF1B	Elongation factor 1-beta	0.074	10.02	0.045	9.86	0.029	10.25
PYT21544	GAPDH	Glyceraldehyde-3-phosphate dehydrogenase	0.230	7.66	0.083	9.92	0.026	10.26
PYT03203	RPL16	39S ribosomal protein L16	0.096	6.32	0.155	4.86	0.061	6.61

**Fig. 3:**
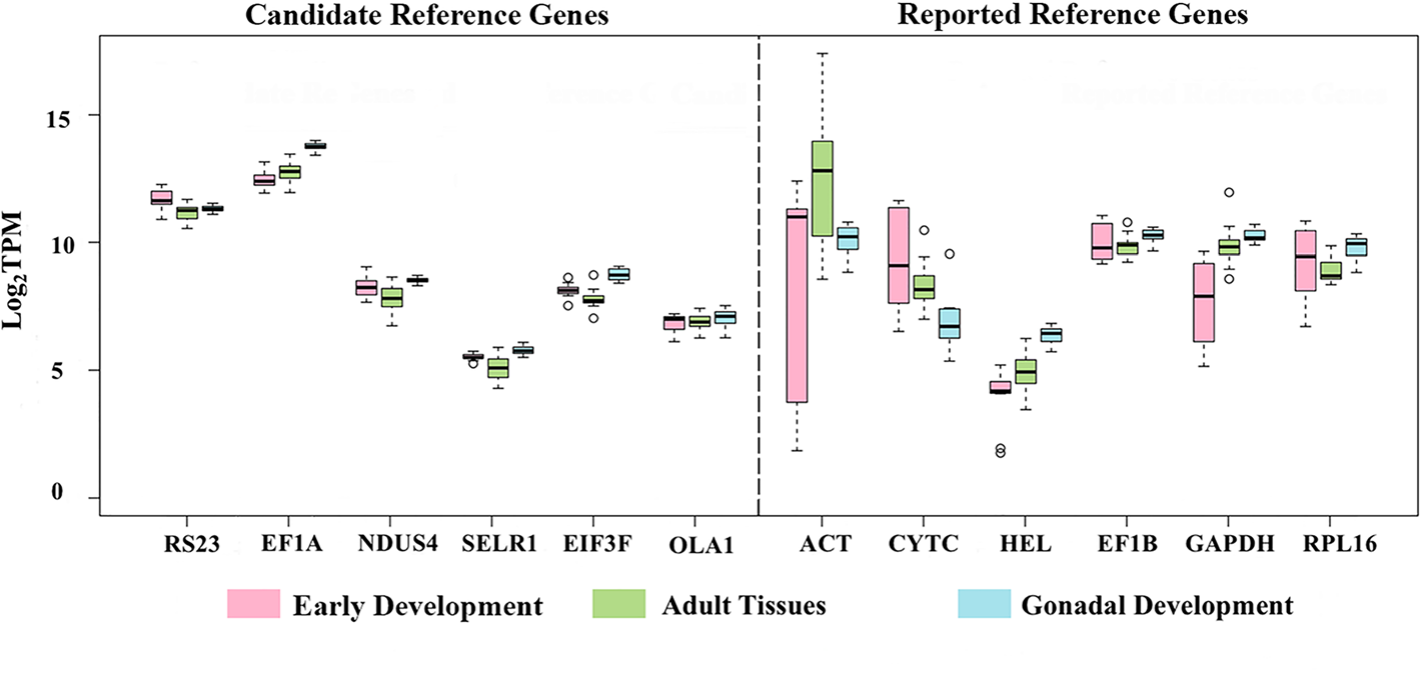
Evaluation of the reference gene candidates and reported reference genes based on RNA-seq analysis. A boxplot showing the log_2_(TPM) values of the 6 candidate reference genes and 6 reported reference genes in early development (pink), adult tissues (green) and gonadal development (blue)

The log_2_(TPM) values of the twelve genes in the three RNA-Seq datasets are shown in Fig. 3. The six candidate reference genes have smaller variances in contrast to the commonly used reference genes in all three datasets, suggesting that these genes are more stably expressed, irrespective of developmental stages or tissue types. The variances differ among datasets for the commonly used reference genes. Most of these genes, including *ACT*, *CYTC*, *GAPDH* and *RPL16*, are highly unstable during early development. In adult tissues, the expression of *ACT* is most variable, and during gonadal development, *CYTC* is most unstable. *EF1B* is relatively stable compared to the other commonly used reference genes in all three datasets, and the only gene that falls within the core set candidate reference gene list.

**Fig. 4:**
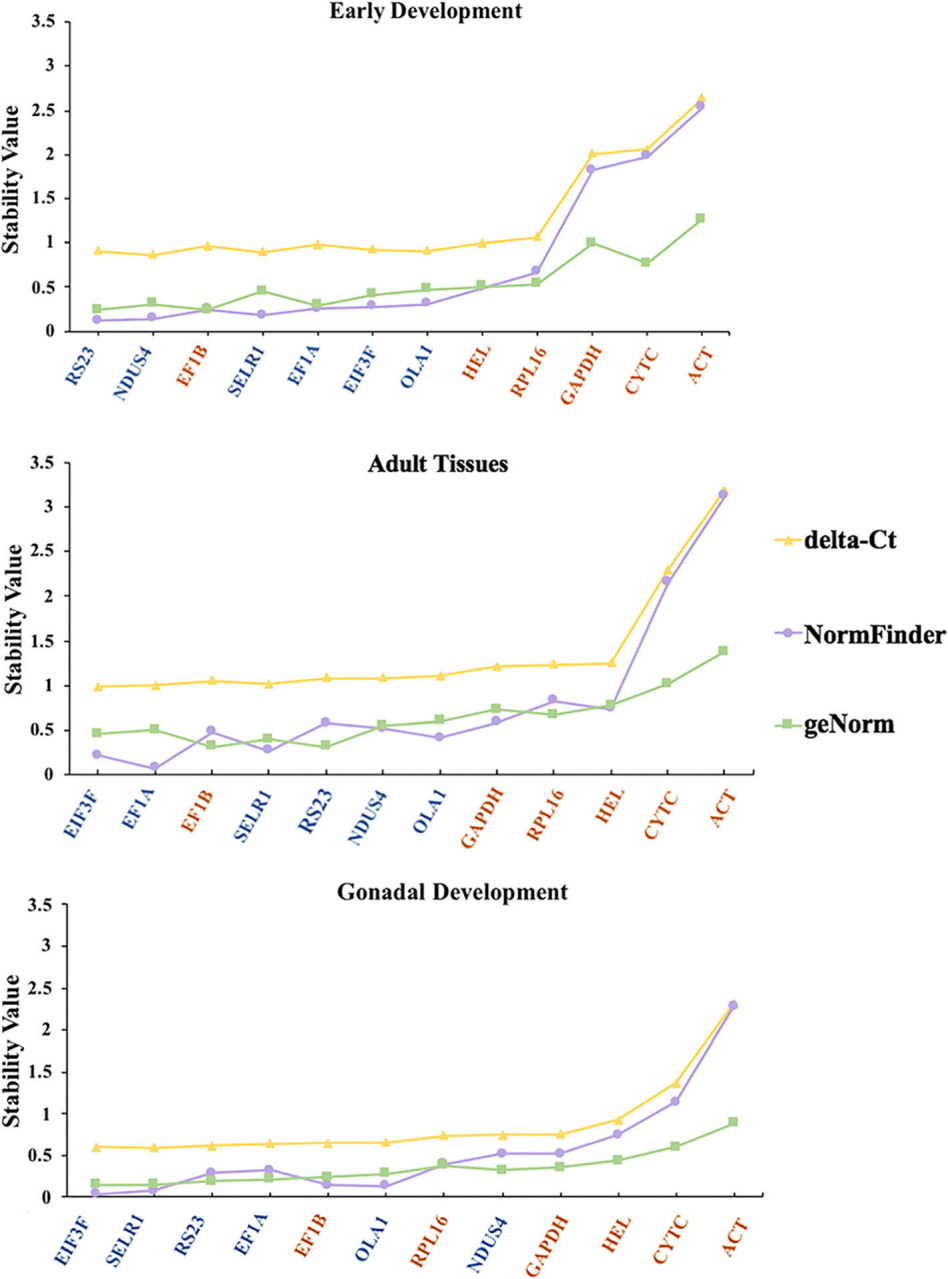
Expression stability of the twelve genes in early development (**a**), adult tissues (**b**) and gonadal development (**c**) based on RT-qPCR experiments. The stability was evaluated based on geNorm, NormFinder and comparative delta-Ct analyses of the RT-qPCR data. The genes are arranged in descending order of comprehensive stability from left to right

To validate the results, RT-qPCR was conducted, and the expression stability of the twelve genes was evaluated using three methods (geNorm, NormFinder, and comparative delta-Ct). As shown in Fig. 4, the three methods gave very similar results for the unstable genes. Specifically, three genes, including *ACT*, *CYTC* and *GAPDH*, are relatively unstable during early development. Two genes, *ACT* and *CYTC*, are unstable in adult tissues. These two genes are also unstable during gonadal development, with *ACT* exhibiting higher expression variation than *CYTC*. Although stability rankings are different among methods for the highly and medium stable genes, the candidate reference genes tend to have higher comprehensive ranking values than those of the commonly used reference genes. Among the twelve genes, the most stable gene for early development is *RS23*, and *EIF3F* is the most stable in adult tissues and during gonadal development.

### Relationship between the Ct of RT-qPCR and the TPM of RNA-Seq

**Fig. 5:**
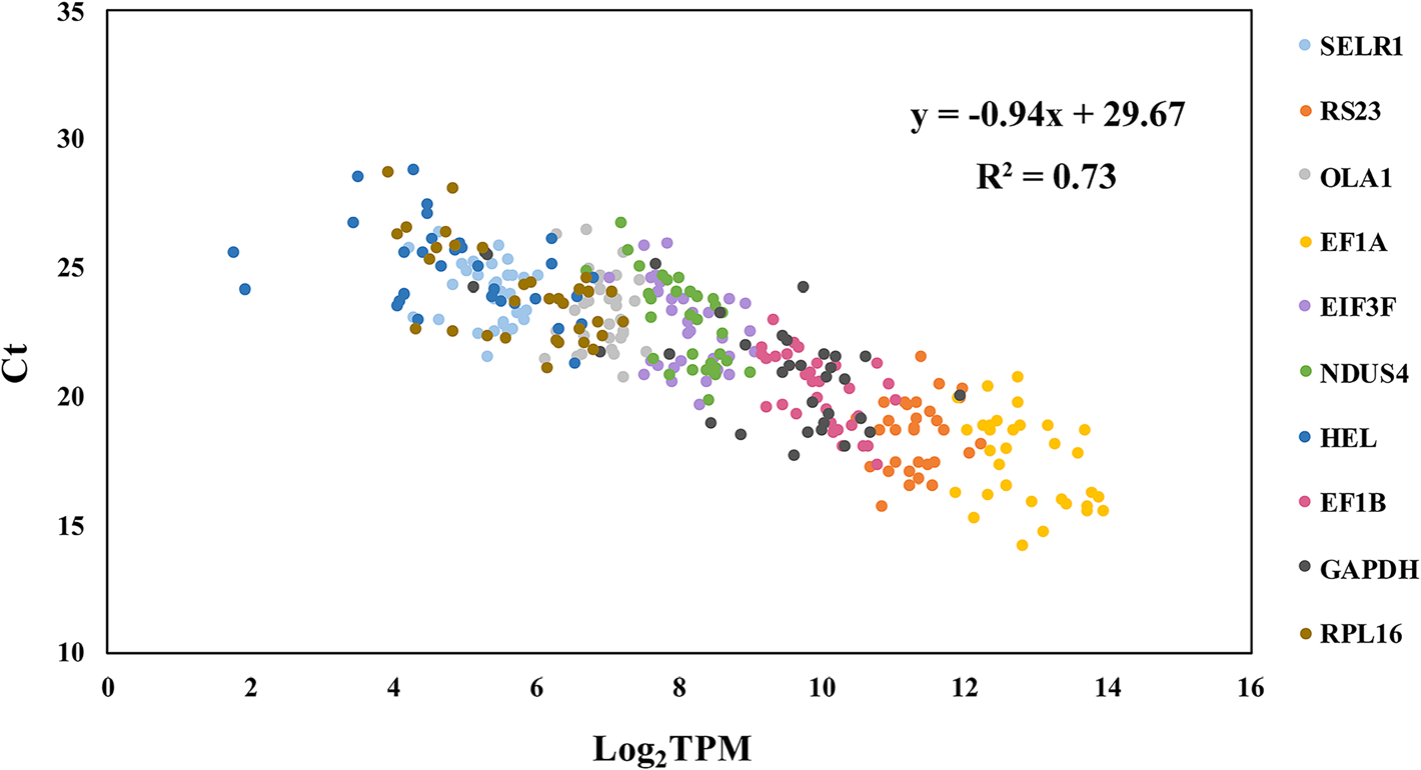
Gene expression correlation between RT-qPCR and RNA-Seq data

To facilitate an estimation of the Ct of RT-qPCR based on RNA-Seq data, we further evaluated the relationship of the gene expression levels between RNA-Seq and RT-qPCR data using the 10 reference genes, excluding *ACT* and *CYTC*. As shown in Fig. 5, the log_2_(TPM) values mainly range from 4 to 14, and the Ct values of RT-qPCR fall between 14 and 29. There is a significantly negative correlation between the two values (r = − 0.85, *P* < 0.01), with the formula Ct = − 0.94 log_2_(TPM) + 29.67.

## Discussion

In this study, an improved method for the systematic identification of reference genes is proposed. Compared with the study by Eisenberg and Levanon [12], there are two modifications in the reference gene screening procedures. First, we used TPM instead of RPKM for gene expression measurement because the widely used RPKM measures can differ substantially between samples and thus potentially cause inflated statistical significance values. In contrast, TPM, as a slight modification of RPKM, eliminates this inconsistency and is suitable for the comparison of RNA abundance among samples [45]. Second, the method provided by Eisenberg and Levanon [12] is developed for the identification of housekeeping genes, but here we aimed to identify reference genes that can be easily detected in RT-qPCR assays. Therefore, the medium to high expression level is used as the fourth criterion. Notably, together with the requirement of low variance among samples (criterion II), the candidate reference genes will have a CV values within 0.2 in our case, which agrees with the standard for reference gene selection in other studies [14, 15, 51]. After applying the four criteria, we obtained candidate reference genes from three separate RNA-Seq datasets and found that the number of candidate reference genes differs among datasets (gonadal development > adult tissues > early development). Further analysis reveals that steps 2 and 4 are the most stringent, which filter out approximately 60~75% of the genes from the previous step. Step 3 is relatively loose and retains 84~97% of the genes. The stringency of step 1 differs among datasets, with a screening rate of 37% for early development, 54% for adult tissues and 62% for gonadal development, indicating that step 1 is the main reason for the difference in the reference gene number among datasets. As criterion 1 requires ubiquitous expression across all samples, the difference in reference gene numbers among datasets can be explained by the variation of sample heterogeneity, i.e., samples of early development are more distinct than those from different tissues or gonadal development. This result is expected because during early development, dramatic changes in gene expression can occur in a short span of time, which has been well reported in other organisms [52, 53]. The four criteria reported in our study facilitate the efficient detection of reference genes from RNA-Seq data.

Functional analysis of the candidate reference genes gives some interesting results. The top 10 most stable genes for adult tissues and gonadal development are all annotated proteins. Although the two gene lists only share a single gene (*RS23*), most of these genes are involved in protein synthesis. In contrast, 4 of the top 10 most stable genes for early development are unannotated. This result is not expected because housekeeping genes are supposed to play important roles in the maintenance of basal cellular functions, and most of these genes are well studied. We assume these unannotated genes in the top stable gene list could be novel genes that are critically important for the early development of scallops, bivalves or mollusks but not for other animals. Among the six annotated genes, four are related to energy production. This result is different from the function of the top reference genes for adult tissues and gonadal development, suggesting that embryos/larvae and adults may emphasize different biological processes or pathways. Consistent with the function of the top stable genes, the 568 core set reference genes are enriched in GO terms and KEGG pathways related to ribosomes, energy production, etc. Similar results are also reported for housekeeping or reference genes identified by genome-wide analysis in other organisms, such as humans [54], mice [13], and maize [55]. These processes should be critically important for life maintenance in various organisms.

The results of RNA-Seq were validated by RT-qPCR for 6 candidate and 6 commonly used reference genes. Although the samples used in RT-qPCR differ from those used in RNA-Seq, there seems to be high consistency between the two results, with candidate reference genes being more stable than most of the traditionally used reference genes. *EF1B* is more stable than the other five commonly used reference genes in early development, adult tissues and gonadal development, which is expected because *EF1B* is in our candidate reference gene list. *ACT* is the least stable gene in early development and adult tissues, which is consistent with the results from a previous report in the same species [29]. However, there is also some inconsistency between our study and the previous study. For example, *CYTC*, which showed medium stable expression in adult tissues in the previous study, is not recommended based on our analysis. This result could be due to a difference in the number of tissues investigated, 6 in the previous study and 11 in our study. The stable expression of *CYTC* and *GAPDH* during early development reported by Feng et al. also deviates from our result, primarily because Feng et al. only evaluated the expression stability until D-stage larvae, but there is obvious *CYTC* downregulation and *GAPDH* upregulation thereafter (Additional file 3: Figure S1). Similarly, the three traditionally used reference genes (*ACT, CYTC and GAPDH*) have been widely reported to be unsuitable as internal controls for RT-qPCR in various organisms, such as oysters, salmon, mice, and humans [32, 56, 57].

For bivalves of economic importance, some research has been conducted on genes related to reproduction [58–60]. Examining their expression dynamics during gonadal development is an important task in these studies. However, several traditionally used reference genes, such as *EF1A* [36, 40, 61], *ACT* [62] and *GAPDH* [63] have been applied, and until now, no study on the transcriptome-wide identification of reference genes in bivalves has been reported. According to our results from RNA-Seq analysis and RT-qPCR validation, *ACT* is not an appropriate reference gene and may result in misleading conclusions. Based on RT-qPCR analysis, *EF1A* ranks fourth after *EIF3F*, *SELR1* and *RS23*, indicating that these genes are all suitable reference genes. The four genes exhibit different expression levels, with *EF1A* being most abundant, followed by *RS23*, *EIF3F* and *SELR1*; thus, researchers can select a reference gene according to the expression level of the genes of interest.

Although a high correlation between RNA-Seq data and RT-qPCR results has been reported in previous studies [36] [64], there is no formula for the relationship between the Ct of RT-qPCR and the TPM of RNA-Seq. The formula presented in our study will facilitate an estimation of the Ct prior to RT-qPCR experiments based on RNA-Seq data in Yesso scallop and might be applied to other organisms.

## Conclusions

In this study, by taking advantage of 60 transcriptomes of the Yesso scallop, we identified candidate reference genes for early development, adult tissues, and gonadal development and found a core set of 568 reference genes. We also conducted stability comparisons of 6 candidate and 6 commonly used reference genes based on RNA-Seq data and RT-qPCR validation and found several traditionally used reference genes that were inappropriate as internal controls. Our study will benefit gene expression studies in bivalve mollusks.

## Additional files

Additional file 1: **Table S1.** Detailed information on the RNA-Seq datasets (XLSM 9 kb)

Additional file 2: **Table S2.** Detailed information on the expression variability and annotation of the candidate reference genes in early development, adult tissues and gonadal development of the Yesso scallop (XLSM 330 kb)

Additional file 3: **Figure S1.** The expression levels of CYTC and GAPDH during the early development of the Yesso scallop (PDF 33 kb)

## Abbreviations

ACT: Beta-actin
ATP5J: ATP synthase-coupling factor 6
ATP5L: ATP synthase subunit g
ATPK: Putative ATP synthase subunit f
CV: Coefficient of variation
CYTC: Cytochrome c
EF1A: Elongation factor 1-alpha
EF1B: Elongation factor 1-beta
EIF3F: Eukaryotic translation initiation factor 3 subunit F
GAPDH: Glyceraldehyde-3-phosphate dehydrogenase
GO: Gene Ontology
HEL: DEAD-box RNA helicase
IF4A1: Eukaryotic initiation factor 4A-I
KEGG: Kyoto Encyclopedia of Genes and Genomes
NDUAB: NADH dehydrogenase [ubiquinone] 1 alpha subcomplex
NDUS4: NADH dehydrogenase [ubiquinone] iron-sulfur protein 4
OLA1: Obg-like ATPase 1
RLA1: 60S acidic ribosomal protein P1
RM54: 39S ribosomal protein L54
RPKM: Reads Per Kilobase Million
RPL16: 39S ribosomal protein L16
RS23: 40S ribosomal protein S23
RT-qPCR: Reverse transcription quantitative PCR
SELR1: Sel1 repeat-containing protein 1
TPM: Transcripts per million
