# Supplementary figures and images for: Systematic identification and validation of the reference genes from 60 RNA-Seq libraries in the scallop Mizuhopecten yessoensis
Source: BMC Genomics. 2019 Apr 11;20:288. doi: 10.1186/s12864-019-5661-x (PMC6460854; doi:10.1186/s12864-019-5661-x)

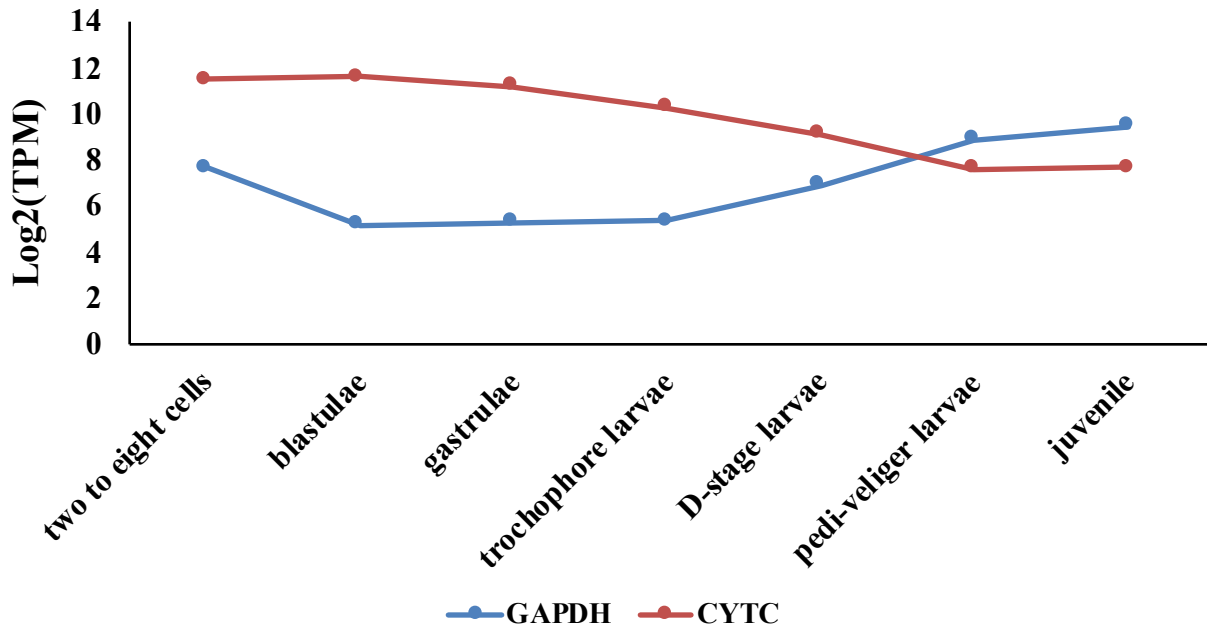

Supplement: Supplementary file 3 — Figure S1. The expression levels of CYTC and GAPDH during the early development of the Yesso scallop (PDF 33 kb) [file 12864_2019_5661_MOESM3_ESM.pdf]
